# Preservation of circadian rhythm in hepatocellular cancer

**DOI:** 10.1016/j.jbc.2023.105251

**Published:** 2023-09-14

**Authors:** Yanyan Yang, Ashraf N. Abdo, Hiroaki Kawara, Christopher P. Selby, Aziz Sancar

**Affiliations:** Department of Biochemistry and Biophysics, School of Medicine, University of North Carolina at Chapel Hill, Chapel Hill, North Carolina, USA

**Keywords:** circadian, hepatocellular cancer, XR-seq, rhythmic in tumor

## Abstract

Circadian rhythms are controlled at the cellular level by a molecular clock consisting of several genes/proteins engaged in a transcription–translation–degradation feedback loop. These core clock proteins regulate thousands of tissue-specific genes. Regarding circadian control in neoplastic tissues, reports to date have demonstrated anomalous circadian function in tumor models and cultured tumor cells. We have extended these studies by analyzing circadian rhythmicity genome-wide in a mouse model of liver cancer, in which mice treated with diethylnitrosamine at 15 days develop liver tumors by 6 months. We injected tumor-bearing and control tumor-free mice with cisplatin every 2 h over a 24-h cycle; 2 h after each injection mice were sacrificed and gene expression was measured by XR-Seq (excision repair sequencing) assay. Rhythmic expression of several core clock genes was observed in both healthy liver and tumor, with clock genes in tumor exhibiting typically robust amplitudes and a modest phase advance. Interestingly, although normal hepatic cells and hepatoma cancer cells expressed a comparable number of genes with circadian rhythmicity (clock-controlled genes), there was only about 10% overlap between the rhythmic genes in normal and cancerous cells. “Rhythmic in tumor only” genes exhibited peak expression times mainly in daytime hours, in contrast to the more common pre-dawn and pre-dusk expression times seen in healthy livers. Differential expression of genes in tumors and healthy livers across time may present an opportunity for more efficient anticancer drug treatment as a function of treatment time.

The mammalian circadian clock coordinates biological functions with the environmental 24-hour light/dark cycle (1). At the molecular level, the clock is driven by a transcription–translation–degradation feedback loop consisting principally of core clock transcriptional activators and repressors (2, 3, 4). These clock proteins control their own daily rhythms of expression via interactions at E-box elements in the promoters of clock genes (5, 6, 7, 8, 9). Clock proteins also control the rhythmic expression of thousands of downstream circadian-controlled genes with E-box promoter elements. Physiologically, the suprachiasmatic nucleus (SCN) functions as a “central pacemaker”; the clocks in SCN cells are entrained to the light/dark cycle mainly by photic input from the eyes; furthermore, signals from the SCN entrain the rhythms of the core clock genes/proteins in cells throughout the body (1, 2, 3, 10). Core clock proteins in cells throughout the body in turn control the rhythms of organ-specific circadian controlled genes. Synchrony in peak and trough core clock gene expression times between the SCN and organs is not perfect; notably, feeding time may also serve as an entrainment cue, causing circadian phase advances or delays in organs such as the liver, depending upon the feeding schedule (11, 12, 13).

Neoplasms by definition exhibit autonomy from host organs to varying degrees (14, 15). The maintenance of circadian rhythms in tumors or the lack thereof is an interesting topic with respect to cancer biology, and to the possible use of chronotherapy in the treatment of cancer, that is, the employment of defined treatment schedules to improve efficacy and/or reduce side effects (16, 17). Studies of circadian clocks in tumor cell lines, organotypic cultures, and xenograft models have yielded various findings, ranging from cancer tissues being arrhythmic, to being rhythmic and either in phase or out of phase with the relevant host organ (16, 18, 19, 20, 21, 22, 23). These studies offer valuable insights, but many also have limitations, for example, they were done with target cells separate from a host setting, or they were done within a host, but measured only a limited number of reporter genes. Further investigation is needed to more comprehensively understand rhythmicity and its dysregulation in tumors.

In this study, we examined rhythmicity in a mouse model of hepatocellular carcinoma. In this model, mice are injected with diethylnitrosamine (DEN) one time, at 15 days of age, and by 25 weeks of age, essentially all mice develop liver tumors (14, 15). To sensitively measure gene expression in these tumors, genome-wide, we used the XR-seq assay to map locations where DNA repair occurs (24). Due to the transcription-coupled repair pathway of excision repair, the template strand of actively transcribed DNA is repaired at an accelerated rate, and in this assay, active transcription is reliably detected as the elevated transcribed strand/nontranscribed strand (TS/NTS) repair ratio in genes (25). XR-seq has been used to thoroughly characterize rhythmic gene expression following injection of healthy mice with the DNA-damaging anticancer drug cisplatin (19, 26), and in this study, we measured gene expression as transcription-coupled repair following injection of tumor-bearing mice with cisplatin over a 24-h period. Our results overall demonstrate tumor gene rhythmicity which remains to a degree in synch with the host but also exhibits significant differences compared to rhythmicity in healthy control mouse livers. In tumors, some of the clock genes expressed robust rhythmicity, with a possible, modest advance in phase compared to healthy liver. Regarding the downstream genes that are circadian-controlled in healthy liver, most of these genes in the tumor were not rhythmic, some were rhythmic but out of phase with expression in healthy liver, and a few were rhythmic and in phase. Surprisingly, many of the genes that are rhythmic in tumors did not exhibit circadian rhythmicity in healthy livers, demonstrating an apparent gain of circadian function in the tumors. Thus, our findings reveal a remarkable complexity in the rhythmic behavior of tumors in the model employed.

## Results

### Mouse model of hepatocellular carcinoma

**Figure 1:**
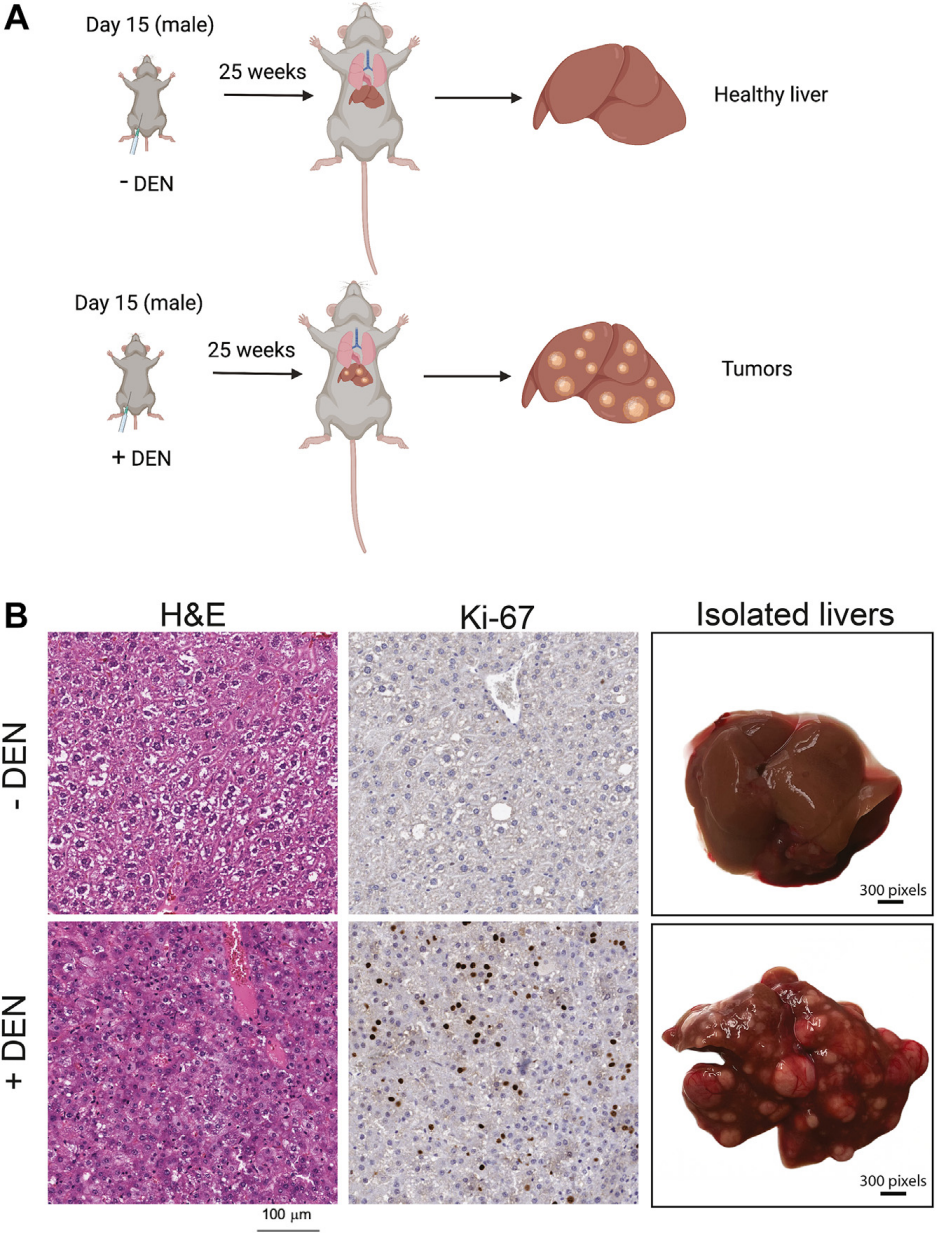
**Mouse hepatocarcinoma model**. *A*, 15-day-old male C3H/HeOuJ mice were injected one time with DEN. This results in tumors by 25 weeks. *B*, histology of healthy liver and tumors and macroscopic view of livers. H&E staining demonstrates tissue morphology, and Ki-67 immunostaining identifies proliferating cells. Scale bar = 100 μm.

We used a well-established mouse model of hepatocellular carcinoma (Fig. 1*A*) in which 15-day-old male C3H/HeOuJ mice injected with DEN uniformly develop tumors by 25 weeks post-injection. All of the mice that we injected with DEN developed multiple, macroscopically identifiable tumors (Fig. 1*B*). Histochemical results in Figure 1*B* demonstrate altered tissue morphology in H&E stained sections from +DEN livers, and show that the Ki-67 proliferation marker (27) is present in tumors and absent in healthy tissue, as previously reported (14). The tumor in the specimen shown appears to represent hepatocellular carcinoma.

### Tumors exhibit transcription-coupled repair

**Figure 2:**
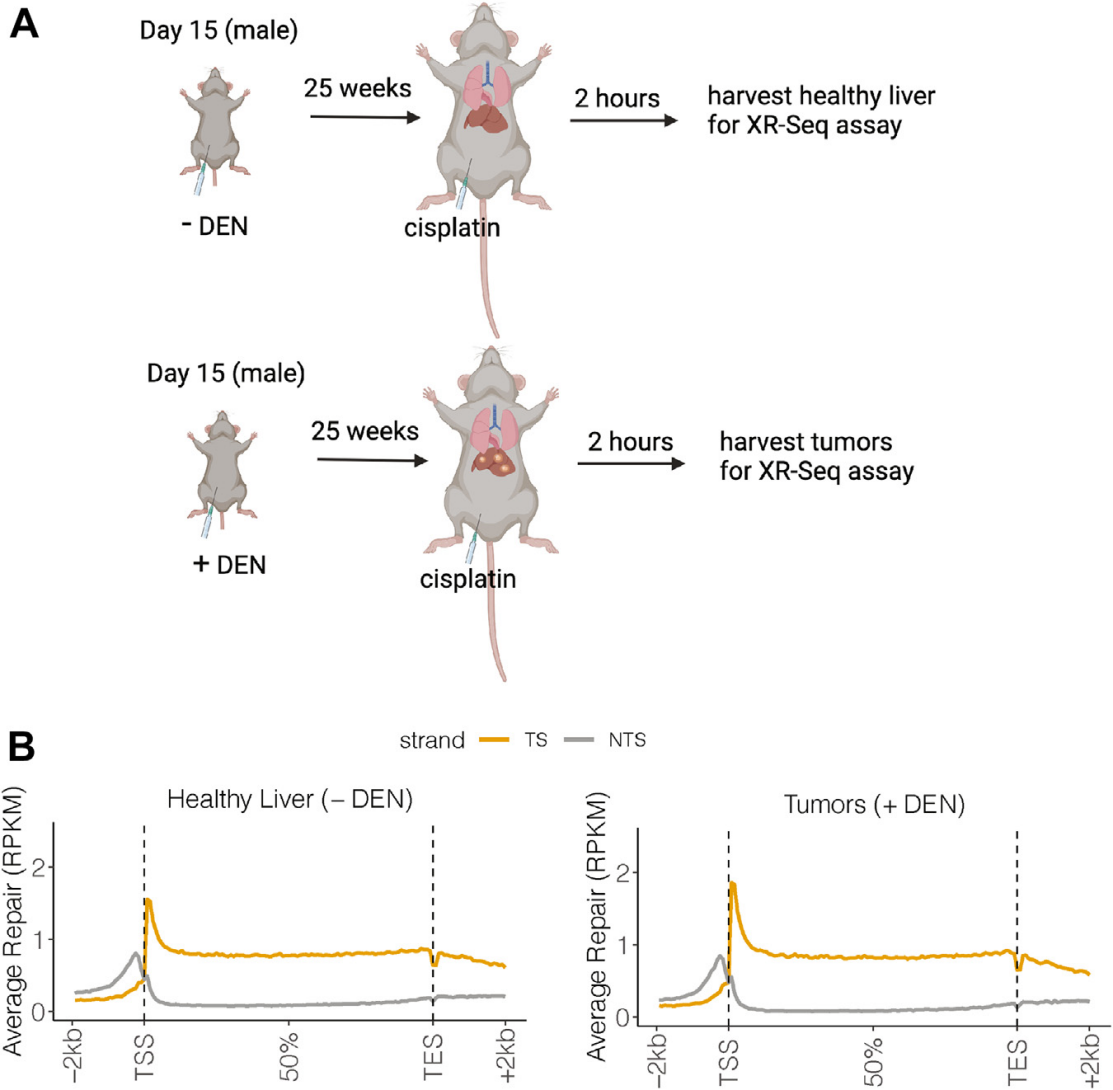
**Transcription-coupled repair in healthy liver and tumors.***A*, schematic of the experiment to analyze TCR. Following injection with DEN at 15 days of age, by 25 weeks mice have liver tumors, while mice not treated with DEN have healthy livers. Healthy and tumor-bearing mice are then injected one time with cisplatin, and following 2 h for repair, healthy livers and tumors are harvested for XR-seq assay. *B*, TCR in liver and tumors. XR-seq reads for all genes were binned and scaled to a unit size and bin average values for the TS (*orange*) and NTS (*gray*) strands are plotted. Results for the average repair signals 2 kbp upstream of transcription start sites (TSSs) and downstream from transcription end sites (TESs) are also plotted. TCR is evident in healthy liver and tumor tissues. RPKM, reads per kilobase pair per million total reads.

We measured genome-wide transcription indirectly as transcription-coupled excision repair of cisplatin-DNA adducts. The enhanced initial rate of TS repair compared to the NTS (due to the enhanced repair rate of transcription-blocking TS lesions) is reliably measured by the XR-seq assay, in which the nominal 26-nt repair products of dual incision are isolated, sequenced, and mapped to the genome. For our experiments, as shown in Figure 2*A*, mice with liver tumors (+DEN) and healthy, tumor-free control mice (-DEN) were injected with cisplatin, and following 2 h to allow for repair, were sacrificed. For XR-seq assay, liver was isolated from healthy (−DEN) mice (referred to in this report as “healthy liver”), and tumors (+DEN) (referred to in this report as “tumors”) were separated from +DEN mouse livers that possessed different sizes of tumors (Fig. 1*B*). For both groups, excision repair product reads were mapped to the genome and further processed to assess the reads within annotated genes. In Figure 2*B*, XR-seq results are plotted by scaling and averaging the results for all genes from healthy liver (left), and tumor (right), from the transcription start sites (TSSs) to the transcription end sites (TESs). Average repair in the 2 kbp regions upstream and downstream are also plotted. Importantly, repair for the TS (orange) and NTS (gray) are plotted separately. A robust transcription-coupled repair signal (TS>NTS) signal is seen both in healthy liver and tumor. The switch to preferential NTS>TS repair upstream of the gene is consistent with prior XR-Seq results obtained from eukaryotic tissues and cells and is attributed to antisense transcription in the promoter region (18, 24).

### Rhythmicity in liver and tumors: Core clock genes

**Figure 3:**
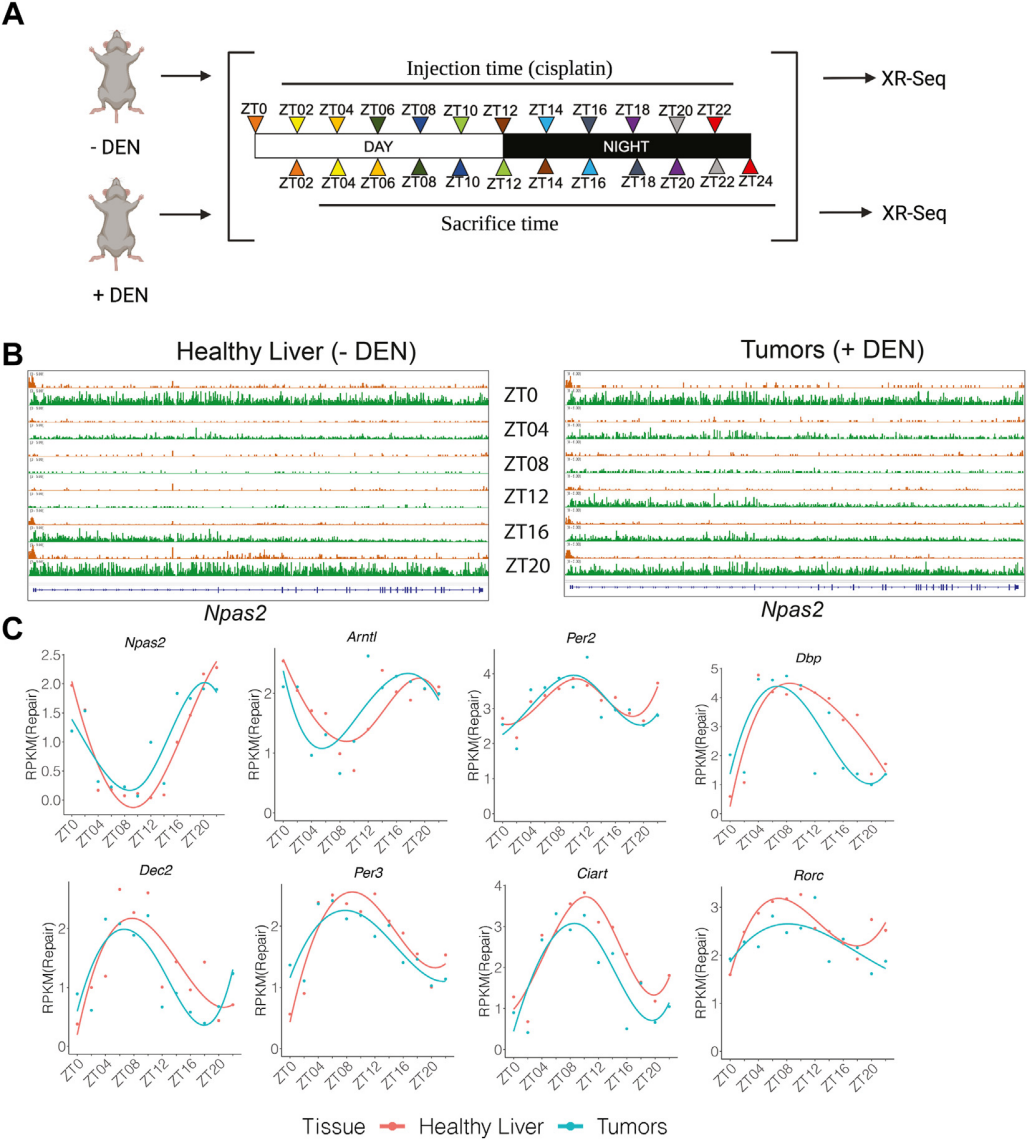
**Rhythmicity in transcription-coupled repair**. *A*, Schematic of the experiment to analyze circadian rhythmicity. Every 2 h over a 24-h cycle, a tumor-bearing (+DEN) and a healthy control mouse (−DEN) is injected with cisplatin. Two hours after each injection, the mouse is sacrificed to analyze repair of cisplatin in healthy liver or tumor by XR-seq. *B*, Screenshot representation of XR-seq results for the clock gene *Npas2* in liver (*left*) and tumors (*right*). Data for 6 of the 12 time points are shown. The gene, indicated with the symbols on the bottom, transcribes from *left to right*, and TS repair is indicated in green, and NTS repair is indicated in orange. Rhythmicity in TS repair is evident throughout the gene (in *green*). Corresponding rhythmicity in the NTS in the region of the promoter (in *orange*) reflects antisense transcription at the promoter. The amount of TS repair in the *Npas2* gene was quantified for all time points and repair values are plotted in the top left panel of (*C*), which illustrates rhythmicity in the expression of *Npas2*. Results for other clock associated genes found to be rhythmic are also plotted in (*C*).

In order to investigate whether tumors have a functional circadian clock, we used XR-seq to compare rhythmicity in gene expression in healthy liver versus tumors, focusing first on clock genes. For this analysis, healthy (−DEN) and tumor-bearing (+DEN) mice were injected with cisplatin every 2 h over a 24-hour period, and then analyzed by XR-seq 2 h after each injection, as depicted in Figure 3*A*. The screenshots in Figure 3*B* show repair of a representative clock gene, *Npas2*, in healthy liver and tumor. At the bottom of the screenshots is a gene schematic; above is shown repair at six of the twelve time points (ZT0, ZT4, ZT8, ZT12, ZT16, and ZT20), plotted as numerous small orange or green bars, which represent the number of individual + and –strand repair reads, respectively. For *Npas2*, the green strand is the TS. As can be seen from the screenshot, a robust circadian rhythm is observed both in healthy liver and tumor. Total TS repair reads for healthy liver and tumor at all time points were quantified and are plotted in Figure 3*C* which illustrates rhythmicity in healthy liver (red) and tumor (blue).

Figure 3*C* also shows quantitative results obtained for other core clock genes found to be rhythmic, including Arntl(Bmal1), and Per2. These and the other clock-related genes in Figure 3*C* exhibit similar amplitudes in both liver and tumor. Interestingly, among these genes, there is a trend for a modest advance in phase by about 2 h in tumor compared to healthy liver. In four cases, this advance is found to be significant (*Naps2*, *p* = 0.021; *Dec2*, *p* = 0.013; *Per3*, *p* = 0.015; *Ciart*, *p* = 0.029) by CircaCompare (28) (Fig. S1*A*). Several other non-clock genes exhibited a similar pattern as illustrated with the examples in Fig. S1*B*.

Other core clock genes, notably *Cry1* and *Nr1d1*, were unexpectedly not identified (as described in Methods) as rhythmic in the healthy liver in this study with C3H/HeOuJ mice by the method employed. This could be due to the repair saturation (29) and mouse strain differences (30).

### Rhythmicity in liver and tumors: Circadian clock-controlled genes

**Figure 4:**
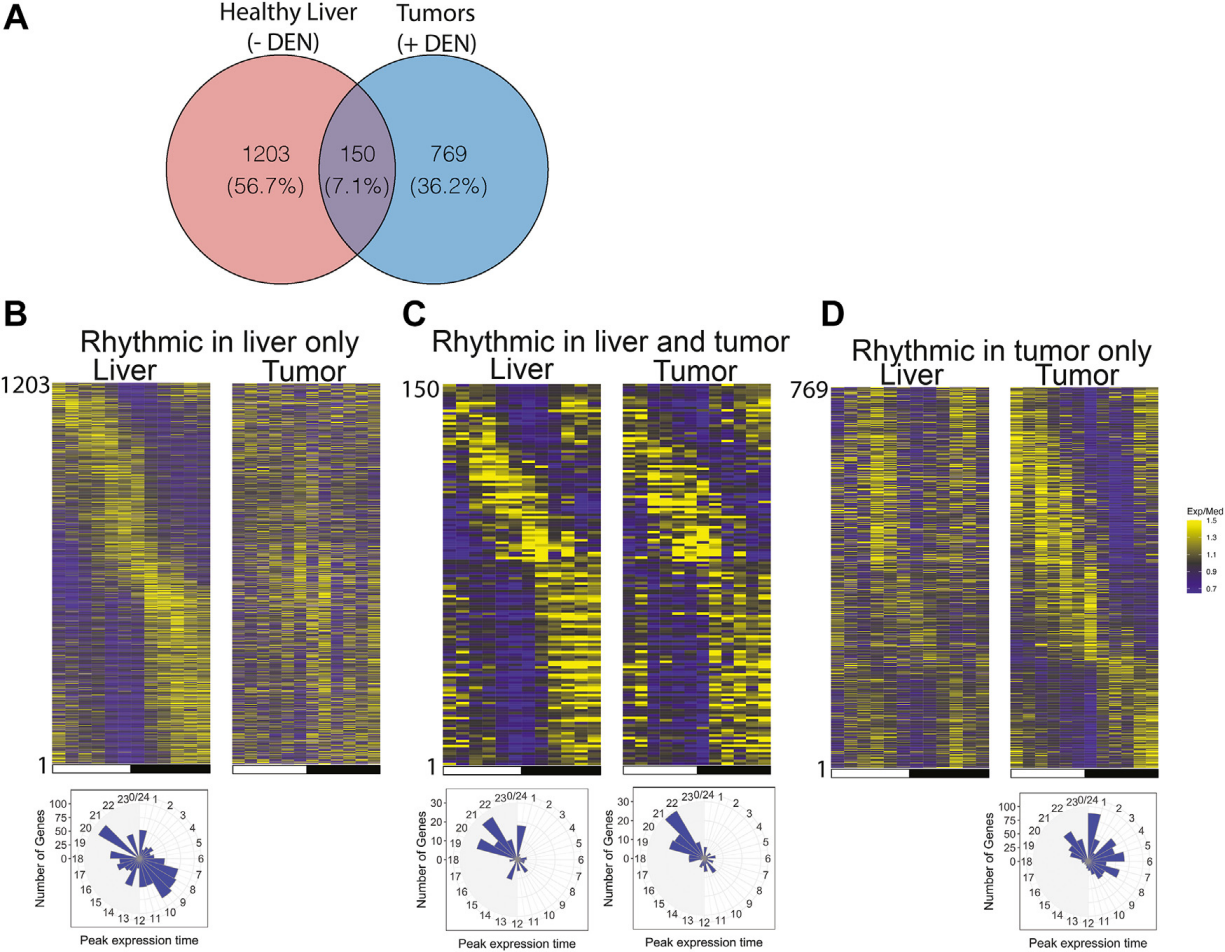
**Comparison of rhythmic genes in healthy liver and tumors**. *A*, Venn diagram illustrating overlap in genes identified as rhythmic in liver and tumors. *B–D*, Heat maps showing the rhythmicity, in both liver and tumor, of genes that are categorized in (*A*) as rhythmic only in liver (*B*), rhythmic in both liver and tumor (*C*), and rhythmic only in tumor (*D*). Below each heat map is an associated radial diagram where appropriate. In the heat maps, the *black/white* bar beneath each indicates *dark/light periods*, and data for the 12 time points are above the bars ordered successively from ZT0 to ZT24 *left to right*. Data for the different genes are ordered from top to bottom based on time of maximal expression (*yellow* is the highest). Relative expression of each gene was determined by plotting, for each time point, the Exp/Med, or the experimental value for each time point divided by the median value for all time points. The radial diagrams illustrate with bars the number of genes with peak expression at each ZT. Background shading in *grey* identifies the *dark hours* between ZT12 and ZT0.

The core clock proteins regulate the rhythmic expression of hundreds of downstream clock-controlled genes in a tissue-specific manner. To investigate these circadian-controlled genes, all rhythmic genes in healthy livers and tumors were identified. The Venn diagram in Figure 4*A* illustrates the concurrence of rhythmic genes in healthy liver and tumor. 150 genes were rhythmic in both healthy liver and tumor tissues, and many of these are associated with circadian rhythmicity as discussed below. An additional set of genes demonstrated liver-specific rhythmicity. Interestingly, a comparable number of genes in tumors also exhibited circadian rhythmicity. However, most of these were tumor-specific.

To examine these three groups of genes in more detail, namely, genes rhythmic in liver and tumor, rhythmic in liver only, and rhythmic in tumor only, each was plotted as a heat map representation in Figure 4, *B*–*D*, with corresponding radial diagrams below where appropriate. In each of the heat map representations, the white/black bar on the bottom indicates the 12-h light and 12-h dark phases. Each of the 12 time point measurements are aligned in succession left to right above the bars, and the different genes are ordered top to bottom based upon their expression time (yellow is highest expression). The plots include the genes identified as rhythmic only in healthy livers (B), genes identified as rhythmic in both healthy livers and tumors (C), and genes rhythmic only in tumors (D). The heatmap and radial diagram in Figure 4*B* (left) show that the genes that are rhythmic only in healthy liver exhibit peak expression times mainly in the pre-dawn and pre-dusk hours, as was previously reported for C57Bl/6 mice (16, 18). The corresponding genes in tumor are not rhythmic and thus a radial diagram is not shown. For the genes rhythmic in both healthy liver and tumor (Fig. 4*C*), pre-dawn peaks predominate. The radial diagrams in Figure 4*C* reveal similarities and differences between peak expression times in liver and tumor, indicating some genes in tumor retain the same phase as in liver, and some are out of phase. Interestingly, among genes rhythmic in tumor only (Fig. 4*D*), many genes exhibit peak expression in the tumor tissue between ZT0 and ZT8, in addition to a more modest pre-dawn peak. Thus, the novel genes rhythmic in tumor only are largely out of phase with most circadian-controlled genes in healthy liver.

### Gene ontology analysis

**Figure 5:**
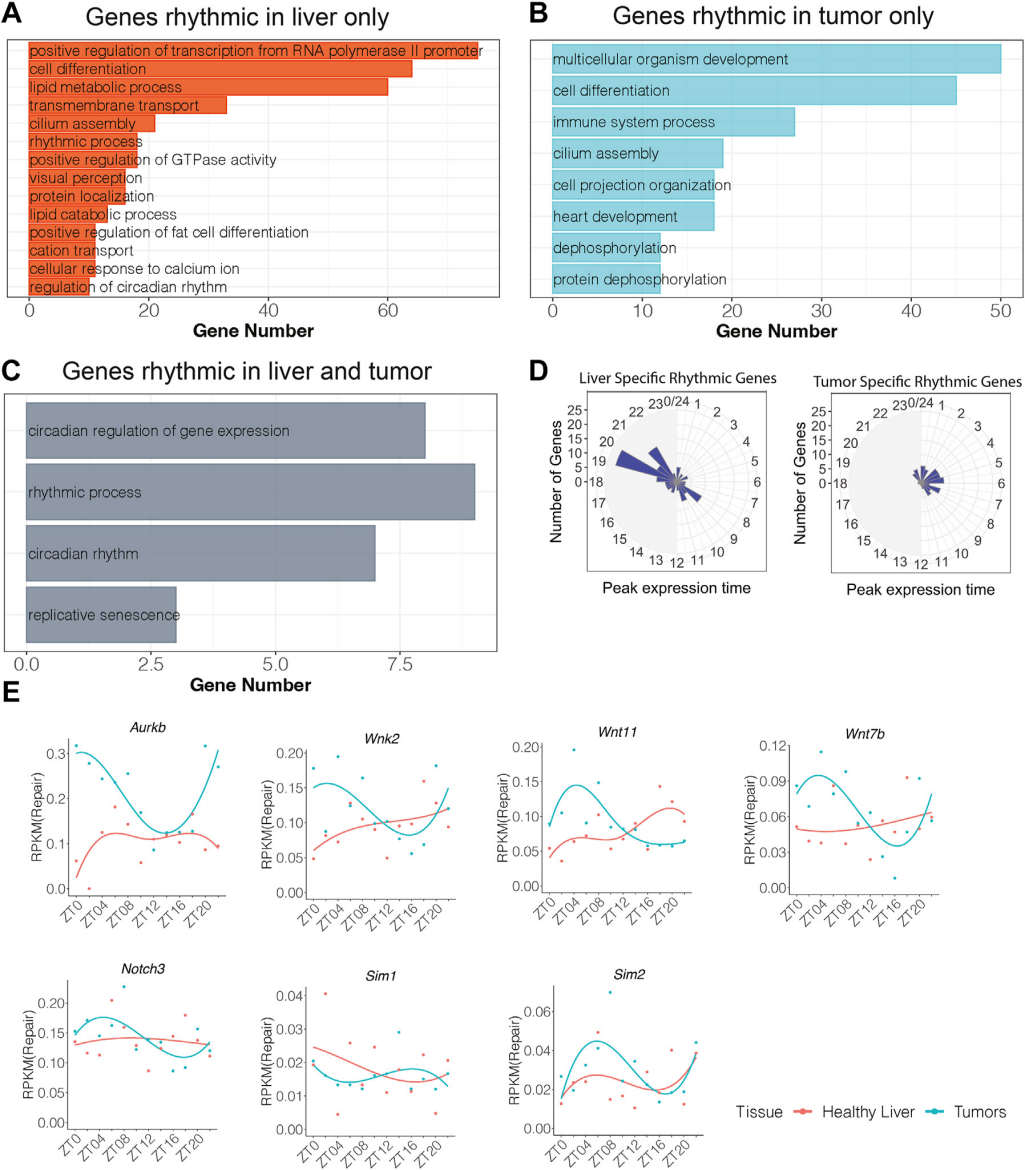
**Gene ontology analysis.** In each plot, categories are listed top to bottom in decreasing abundance, with the number of genes in each category plotted in *red* for genes rhythmic only in healthy liver (*A*), in *blue* for genes rhythmic only in tumor (*B*), and in *grey* for genes rhythmic in both liver and tumor (*C*). *D*, radial diagrams showing rhythmicity of genes involved in regulation of transcription/organism development/differentiation that are rhythmic in liver only (*left*) and tumor only (*right*). *E*, select genes rhythmic in tumors but not healthy liver.

To investigate the biological function of the oscillating genes in both healthy liver and tumors, we used the DAVID functional annotation tool to perform pathway analysis of cycling genes. Results are shown in Figure 5, and the individual genes that are rhythmic in liver only (A), tumor only (B), and rhythmic in both liver and tumor (C) are listed in Table S1. Genes involved in rhythmic processes were relatively abundant in genes classified as “rhythmic in liver only” and “rhythmic in both liver and tumor”. These results are consistent with the finding that tumors retain rhythmicity, albeit different from healthy liver as described above. Genes involved in cell differentiation, multicellular organism development and positive regulation of transcription are found to be relatively abundant among genes that are “rhythmic in liver only” and “rhythmic in tumor only”. These functions are associated with cellular and neoplastic growth, and interestingly, the peak expression times for these genes are different in healthy livers and tumors, as shown in Figure 5*D*. Another notable difference is a set of cancer-associated genes that are significantly rhythmic in tumor (31, 32, 33, 34, 35, 36, 37) only; examples of these are shown in Figure 5*E*. For this class of genes rhythmic in tumor only, we were interested to see if protein expression was rhythmic in tumor only. AURKB was selected as an example of this class, and western blots probing AURKB (Fig. S2) showed that protein rhythmicity was, in fact, evident in tumor but not in healthy liver. Interestingly, while Aurkb gene expression is high at ZT0 and low at ZT12, Aurkb protein expression is the opposite, high at ZT12 and low at ZT0. This is presumably due to delays in full-length RNA transcription, RNA processing, and protein translation and also the relative half life of the Aurkb protein (38).

## Discussion

A number of experimental systems are available to examine carcinogenesis and circadian biology (13, 16, 20, 21, 22, 23, 39, 40). For both areas of study, whole animals provide a setting that includes the many complex interactions that contribute to and maintain the pathological and rhythmic states. For our investigation of the effect of carcinogenesis on circadian rhythmicity, we opted for this holistic approach, using a well-characterized mouse model for hepatic carcinogenesis (14, 15). In this model, at 6 to 10 months post-DEN injection, the time point we employed, liver tumors are present transitioning from dysplastic nodules to carcinoma, and nearby liver tissue exhibits microscopic foci with altered cellular structures that presumably constitute pre-cancerous growths. Thus, the tumors examined in our studies represent an intermediate phase of carcinogenesis.

This study finds that liver tumors retain robust circadian rhythmicity in gene expression. There are similarities but also significant differences in the rhythmic gene expression in tumors and healthy liver. Among the genes that are rhythmic in both tumors and healthy liver, a number are clock genes known to be involved in self-sustained oscillations within the cell, in addition to several clock-related genes. Interestingly, the phase of the rhythmic peaks and nadirs of expression of these clock genes in tumors were slightly advanced compared to the same genes in normal liver. This finding is consistent with prior studies that showed altered phases in circadian gene expression associated with cellular proliferation (41). Other genes rhythmic in both tumor and liver were either in phase or out of phase.

Aside from the clock genes and other genes rhythmic in both liver and tumor, in transitioning to the neoplastic state, many genes apparently became arrhythmic and many became rhythmic. Considering the genes that became arrhythmic, the question arises as to whether these genes that became arrhythmic in tumors actually fail to oscillate, or do the genes oscillate within each tumor cell but with the cells out of sync with each other? Since we observed rhythmicity of core clock and clock-controlled genes in tumors, it is more likely that the loss of rhythmicity in a set of tumor cell genes was due to a failure of these downstream circadian-controlled genes to oscillate within each cell. The reason for this failure is unclear (41). The many circadian-controlled genes are known to have promoters of varying complexity, with multiple regulatory elements, which, under homeostatic conditions, lead to different genes exhibiting peaks and nadirs of expression at different times of the day and in a tissue-specific manner (7). In the case of tumors, it is possible that regulatory elements that gate circadian controlled rhythmicity in certain genes become compromised in the tumors.

Interestingly, the tumors demonstrated a gain of rhythmicity in a set of genes. This could be due to loss of inhibition by a regulator that gates the circadian expression of these genes. Alternatively, the presence of the tumors may alter the physiology of the host leading in turn to changes in tumor gene rhythmicity. To address the latter possibility, an assessment of tumor impact on rhythmicity of host tissues would be informative. It is also possible that the gain in rhythmicity in some tumor genes may not reflect a gain of true circadian control. Certain genes are rhythmically induced not by core clock genes, but directly in response to factors such as feeding and lighting conditions, which may be rhythmic. In healthy mice, ultradian genes with 12-h rhythmicity have been identified, and in some cases, they appear to have different cues for each of the two daily shifts in gene expression (42, 43). In one study, a loss of one of the two daily shifts occurred in association with imposition of restricted feeding, leading to a change from an ultradian, 12-h rhythmicity to an apparent circadian (24-h) rhythm (43). Analogously, some of the genes that we found to gain apparent circadian rhythmicity in tumors may in fact be ultradian in healthy liver but lose one of two induction signals in transitioning to the neoplastic state. In fact, a preliminary analysis of the genes that gained circadian rhythmicity in tumors in this study suggests that 34 of these genes are ultradian, with a 12-h period in the healthy liver (data not shown). As these are a small number of genes, higher resolution studies are needed to examine in more detail possible transitioning of genes from ultradian to apparent circadian rhythmicity during tumorigenesis.

Differences between the rhythmicity of tumors and healthy tissues may provide a basis for chronotherapy. Many currently available anticancer therapies target the proliferative capacity of tumors, so it is notable that we observed liver-tumor differences in the rhythmicity of genes associated with nucleic acid metabolism and thus proliferation. Of the genes, “rhythmic in tumor only” were 95 genes classified as “multicellular organism development” or “cell differentiation.” These included genes associated with carcinogenesis such as Aurkb, Wnk2, Wnt11, Wnt7b, Notch3, Sim 1, and Sim2 (Figs. 5*E* and S2) (31, 32, 33, 34, 35, 36, 37). Importantly, the “rhythmic in tumor only” proliferation genes exhibited peak expression times (ZT0 to ZT8) that differed from the peak expression times of “rhythmic in liver only” nucleic acid metabolism genes (predawn and predusk, Fig. 5*D*). This offers a basis for attempting chronotherapy of the liver tumors in this model.

## Experimental procedures

### Mouse model

C3H/HeOuJ male and female mice were purchased from Jackson Laboratory (Bar Harbor, ME), and maintained on a light: dark 12:12 schedule. ZT24/0 is the time of light-on and ZT12 is the time of light-off. Animal studies were approved by the University of North Carolina School of Medicine (Institutional Animal Care and Use Committee).

P15 male C3H/HeOuJ mice were treated with a single intraperitoneal (IP) injection of DEN (Sigma-Aldrich N0258; 20 mg/kg body weight) diluted in 0.85% saline to stimulate hepatocarcinogenesis. Livers from healthy control (−DEN) mice and +DEN mice were collected 25 weeks after treatment at the earliest. Tumors were resected from livers of DEN-treated mice for analysis. Males were used since they reliably develop liver tumors; females do not efficiently biotransform DEN to the ultimate carcinogen and are not suitable for use (44).

### Cisplatin injection

Cisplatin (each mL contains: 1 mg cisplatin and 9 mg sodium chloride in water for injection, Fresenius Kabi Pharmaceutical company) was administered by IP injection at 10 mg cisplatin/kg.

### Histological analysis

Formalin-fixed paraffin-embedded tissue sections were stained with hematoxylin and eosin (H&E) using standard laboratory techniques at the Histology Research Core Facility UNC (the University of North Carolina at Chapel Hill). Ki-67 (Abcam #ab16667) at a dilution of 1:500 was used for Ki-67 staining. Images were taken at the Pathology Services Core (the University of North Carolina at Chapel Hill) at 20× resolution.

### XR-seq assay

Treatment of mice with cisplatin, harvesting of tissues, and purification of excision products was as described previously (18). In addition, tumors were isolated from surrounding liver tissue for XR-seq assay. Excision products were treated with NaCN to remove Pt prior to PCR. The remaining steps, oligonucleotides, and adaptors were as described previously (26).

### XR-seq analysis

Flanking adapter sequences were removed from the reads using cutadapt (https://cutadapt.readthedocs.io/en/stable/). Duplicate reads were removed by FASTX-Toolkit with command options fastx_collapser -v -Q33. Reads were aligned to the mm10 mouse genome using bowtie2 with command options bowtie2 -f --very-sensitive -x -u -s. Following alignment, the files were split into plus and minus strands for subsequent analysis. The bigwig file is visualized by IGV (45). For plotting average repair profiles as a unit gene, we chose the genes with length > 5 kbp for *Mus musculus*, and the distance between genes was at least 5 kbp. Each gene was evenly divided into 100 bins from the TSS to the TES, the 2 kbp (25 bins) upstream of each TSS and 2 kbp (25 bins) downstream of each TES were also averaged and plotted, and for each bin, from first to last, an average value for all of the selected genes was obtained and plotted. The y axis average reads per kbp per million total reads (RPKM) for each bin was plotted with R.

Aligned reads were strand-specifically assigned to genes using bedtools with command line options bedtools intersect -c -a -b (46). The reference gene list mm10 was downloaded from the University of California Santa Cruz (UCSC) genome browser and was used to remove overlapping genes.

TS gene hit numbers were normalized by Reads Per Kilobase Million (RPKM), and then by meta2d (47) with a default set except minper = 24, maxper = 24 and cycMethod = c("JTK", "LS"). Ultradian genes detected by minper = 12, maxper = 12, cycMethod = c("JTK", "LS"). The limit for selecting the significant cyclical genes is JTK_pvalue < 0.05 (47).

## Data availability

The raw data and alignment data are available on SRA, accession number PRJNA973991.

## Supporting information

This article contains supporting information (28).

## Conflicts of interest

The authors declare that they have no conflicts of interest.

## Supporting information

Table S1

Figure S1 **Compa****rison of phases determined by CircaCompare** (28)**.** *A*, clock genes and (*B*) examples of clock-controlled genes rhythmic in both healthy liver and tumors and exhibiting a modest phase advance in tumor tissue.

Figure S2 **Protein expression****analysis showing AURKB oscillation in tumors but not in a healthy liver.***A*, stain-free gels showing total protein loaded per lane were used to normalize the immunoblot signals for ARNTL and AURKB. *B*, quantification of three independent western blots for BMAL and AURKB. Statistical analysis was done using t-test; mean ± SD (∗∗*p*-value < 0.01) values are shown.
