# Supplementary figures and images for: Preservation of circadian rhythm in hepatocellular cancer
Source: J Biol Chem. 2023 Sep 14;299(10):105251. doi: 10.1016/j.jbc.2023.105251 (PMC10582759; doi:10.1016/j.jbc.2023.105251)

Figure S1

A

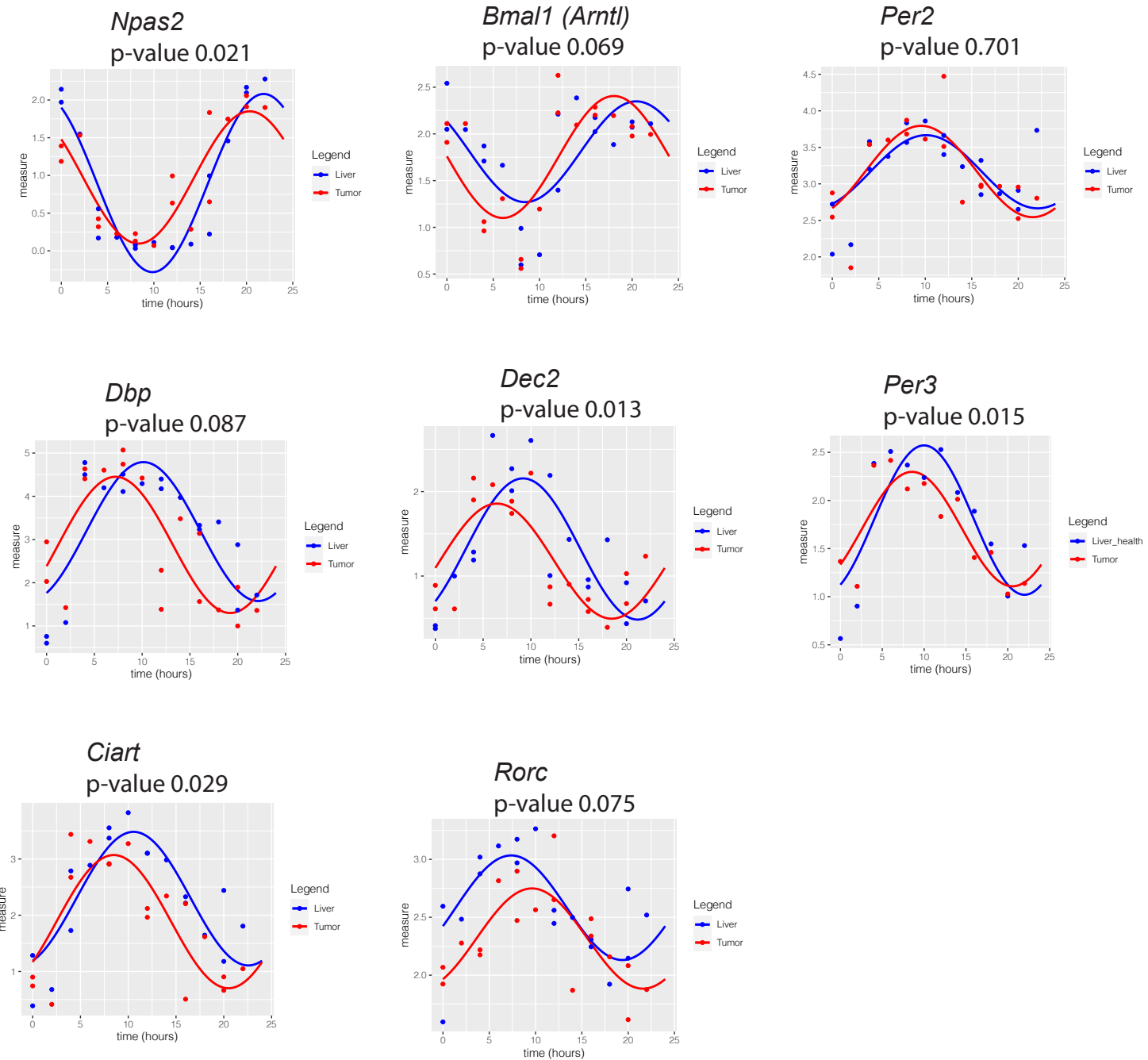

B

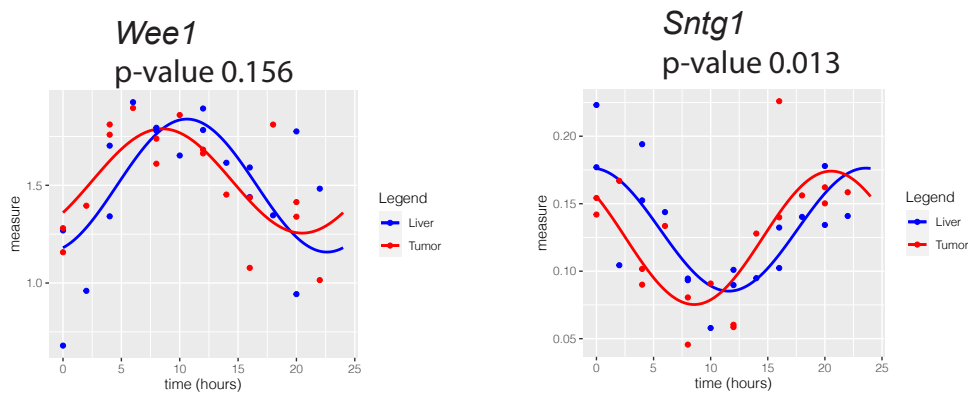

Supplement: Figure S1 — Comparison of phases determined by CircaCompare (28). A, clock genes and (B) examples of clock-controlled genes rhythmic in both healthy liver and tumors and exhibiting a modest phase advance in tumor tissue. [file mmc2.pdf]

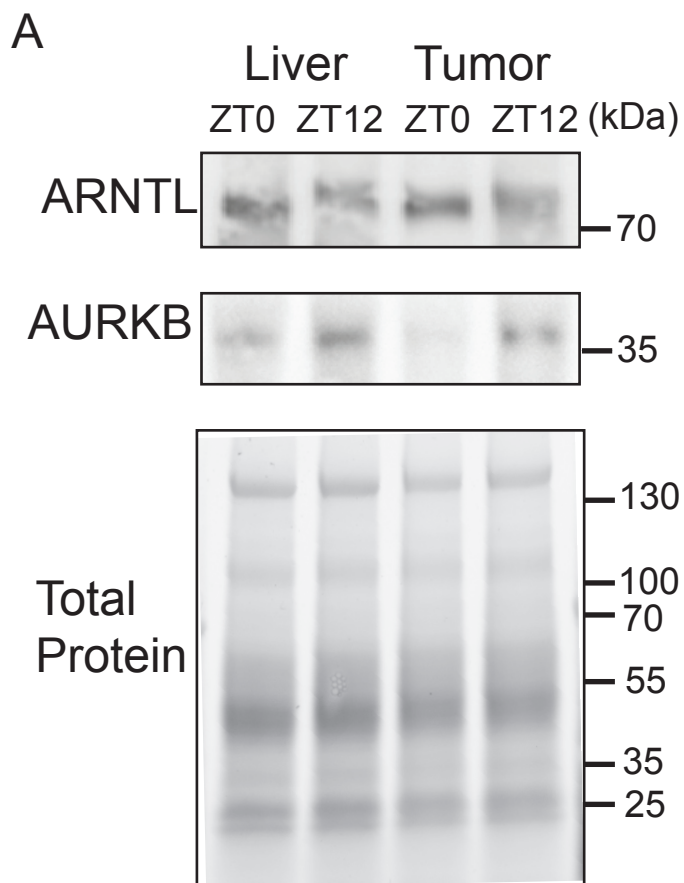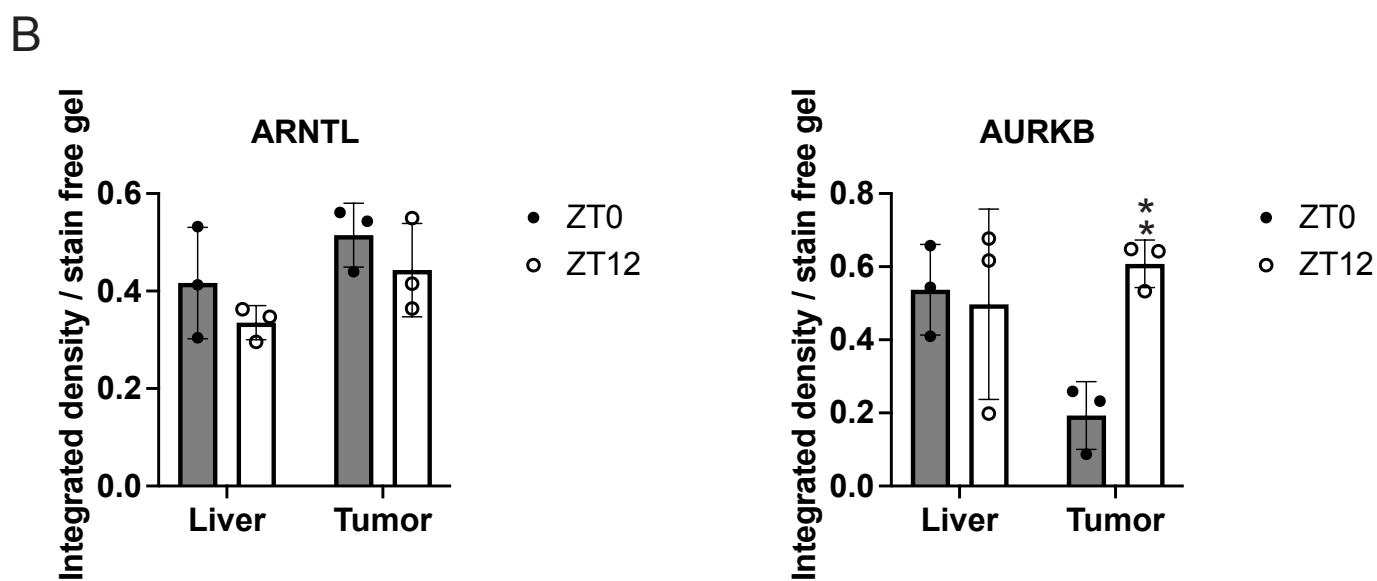

Supplement: Figure S2 — Protein expressionanalysis showing AURKB oscillation in tumors but not in a healthy liver.A, stain-free gels showing total protein loaded per lane were used to normalize the immunoblot signals for ARNTL and AURKB. B, quantification of three independent western blots for BMAL and AURKB. Statistical analysis was done using t-test; mean ± SD (∗∗p-value < 0.01) values are shown. [file mmc3.pdf]
